# A new cryptic *Sympistis* from eastern North America revealed by novel larval phenotype and host plant association (Lepidoptera, Noctuidae, Oncocnemidinae)

**DOI:** 10.3897/zookeys.379.5765

**Published:** 2014-02-12

**Authors:** Brigette Zacharczenko, David L. Wagner, Mary Jane Hatfield

**Affiliations:** 1 Department of Ecology and Evolutionary Biology, University of Connecticut, Storrs, Connecticut 06269-3043, USA; 2 3079 Coldwater Creek Road, Cresco, Iowa, 52136, USA

**Keywords:** Cryptic species, *Triosteum*, Caprifoliaceae, iridoid glycosides, unequal evolutionary rates

## Abstract

A *Triosteum*-feeding species of *Sympistis* is described from eastern North America: *Sympistis forbesi* **sp. n.** Identity of the new species is most reliably determined from larval morphology and host plant association—both adult scaling and genitalic characters overlap with those of *Sympisitis chionanthi*, a *Chionanthus* and *Fraxinus* feeder.

## Introduction

*Sympistis* Hübner is the second largest genus of North American macrolepidopterans, with 176 recognized species (Troubridge 2008, Lafontaine and Schmidt 2010) and many others awaiting formal description. *Sympistis forbesi* sp. n. was first mentioned as a *Triosteum*-feeding variant of *Adita chionanthi* (J. E. Smith) by Rummel (1921) who published a brief account of the larva and its biology. Although Forbes (1954) treated the *Triosteum*-feeder as a “well-marked food strain” of *Adita chionanthi* [now *Sympistis chionanthi*], it is clear that he suspected that the moth represented a valid species, because he provided differential diagnoses for both the adult: “a little less crispy marked, the anal dash a little diffuse, or located in a blackish smudge,” and the last instar: “head green, shaded behind with pale brownish, body yellow-green, the dorsum largely purple-red, with a paler often greenish dorsal line, and a fine white subdorsal near edge of the purple portion; tubercles i and ii small and white, on it. Three dark green lateral lines, the ground usually darkened between the two lower; a broad whitish stigmata line.” Larvae of the two species are figured in Wagner et al. (2011). *Sympistis chionanthi* feeds on *Fraxinus* L., *Chionanthus* L., and perhaps other members of the Oleaceae, whereas *Sympistis forbesi* is believed to be associated only with *Triosteum* L. in the Caprifoliaceae, although larvae can be reared on *Fraxinus* in the laboratory. Given the obvious differences in larval coloration and diet, it is evident that Forbes, and perhaps other noctuoid workers of his day, placed less weight on larval and life history characters than lepidopterists afford them today.

Our motivation for the description of the *Triosteum*-feeding *Sympistis* is that both *Sympistis chionanthi* and *Sympistis forbesi* are worthy, or are likely to become, conservation targets. *Sympistis forbesi* is believed to be extirpated from New Jersey (where Rummel first reported the species) and no extant colonies are known in New York (where Forbes and other Cornell lepidopterists knew it). We think it likely that it is declining or already extinct from much of its former eastern range due to decline in the abundance of *Triosteum* (feverwort), which fared better in the open agricultural landscapes of the two previous centuries. Increased grazing pressure by white-tailed deer is also thought to be a threat to the host plant and its herbivore fauna (Wagner et al. 2011). The only known extant colonies are those present in prairie areas where *Triosteum* is still locally common. *Sympistis chionanthi*, while still widespread across its range (extending from North Dakota to Nova Scotia south to at least Virginia and Kansas), is threatened by the destruction of its primary host by the Emerald Ash Borer (*Agrilus planipennis* Fairmaire) (listed in Wagner 2007).

Below we describe the new *Sympistis*, illustrate the larval, pupal, and adult stages and provide a brief account of the biology of the new species.

## Methods

The adult description of *Sympistis forbesi* is based on 45 pinned specimens from Iowa, Illinois and Minnesota. Seventy-one specimens of *Sympistis chionanthi* from Connecticut and New York were studied (n=71). The larval description of *Sympistis forbesi* is based on 15 preserved larvae and 65 larval images (GGC, ISIC, UCMS). Larvae were compared to 7 preserved larvae and 11 larval images of *Sympistis chionanthi* (CUIC, GGC, NYSM, UCMS). Genitalia of the male type and one female paratype were prepared and mounted according to Lafontaine (2004) except that the preparations were mounted in euparol. Two additional genitalic preparations were left in glycerin. Six slide mounted genitalic preparations made by John G. Franclemont, identified as *Triosteum*-feeding strains of *Sympistis chionanthi*, were borrowed and examined from CUIC. Thirteen *Sympistis chionanthi* genitalic preparations (from New York, Connecticut, Manitoba, Ontario, and Saskatchewan) were examined. COI sequences were generated by the Barcodes of Life Project. Sequences for two *Sympistis forbesi* specimens (Barcodes of Life Project Numbers CNCLEP 81921 and CNCLEP 81922) and six *Sympistis chionanthi* from Ontario and Quebec (Barcodes of Life Project Numbers CNCLEP 81919, CNC Noctuoidea 7959, DH007094, DH009854, 2005-ONT-1897, 2005-ONT-1928) have been deposited at GenBank.

### Abbreviations

CNC Canadian National Collection, Ottawa, Ontario, Canada.

CUIC Cornell University Insect Collection, Ithaca, New York, USA

GGC George Godfrey Collection, Athens, IL, USA

CHC Chuck Harp Collection, Littleton, Colorado, USA

ISIC Iowa State Insect Collection, Iowa, USA

NDSU North Dakota State University, North Dakota, USA

NMNH National Museum of Natural History, Washington D.C., USA

NYSM New York State Museum, Albany, New York, USA

UCMS University of Connecticut, Storrs, Connecticut, USA

## Taxonomy

### Sympistis forbesi

Zacharczenko & Wagner sp. n.

http://zoobank.org/A3005B5D-DCC6-42D1-B743-E1CD7A843763

http://species-id.net/wiki/Sympistis_forbesi

Figs 1 , 2 , 4 , 5 , 7 , 8 , 10 , 11 , 13 , 14 , 18 [Fig F3] [Fig F4] [Fig F5] –32 , 34–36

#### Material examined.

**Figures 1–12.**
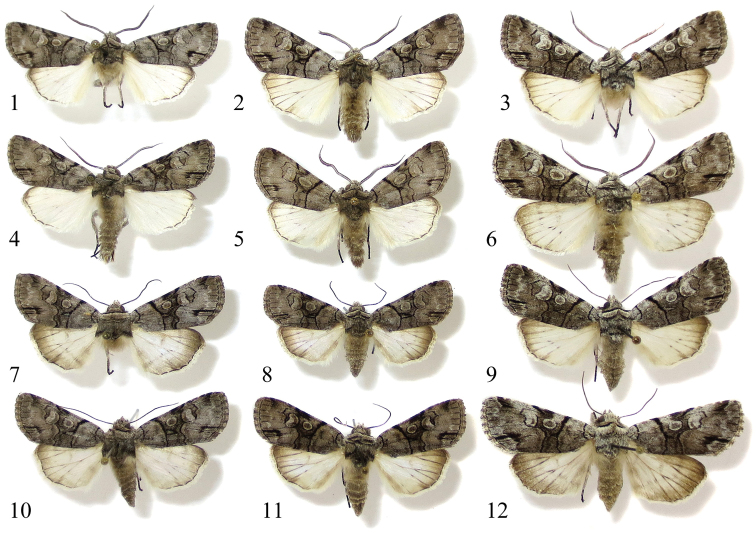
Adults of *Sympistis forbesi* and *Sympistis chionanthi*. **1** ♂ *Sympistis forbesi* HOLOTYPE, IA: Boone Co., Little Blue Stem Prairie, ex larva on *Triosteum* (UCMS) **2** ♂ *Sympistis forbesi*, IL: Champaign Co., Mahomet, ex larva on *Triosteum* (CUIC) **3** ♂ *Sympistis chionanthi*, NY: Tompkins Co., Ithaca, ex ova, reared on *Fraxinus* (CUIC) **4** ♂ *Sympistis forbesi*, IA: Boone Co., Little Blue Stem Prairie, ex larva on *Triosteum* (UCMS) **5** ♂ *Sympistis forbesi*, IL: Champaign Co., Mahomet, ex larva on *Triosteum* (CUIC) **6** ♂ *Sympistis chionanthi*, CT: Windham Co., Hampton, adult at light (UCMS) **7** ♀ *Sympistis forbesi*, IA: Boone Co., Little Blue Stem Prairie, ex larva on *Triosteum* (UCMS) **8** ♀ *Sympistis forbesi*, IL: Champaign Co., Mahomet, ex larva on *Triosteum* (CUIC) **9** ♀ *Sympistis chionanthi*, NY: Tompkins Co., Ithaca, ex ova, reared on *Fraxinus* (CUIC) **10** ♀ *Sympistis forbesi*, IA: Polk Co., ex larva on *Triosteum* (UCMS) **11** ♀ *Sympistis forbesi*, IL: Champaign Co., Mahomet, ex larva on *Triosteum* (CUIC) **12** ♀ *Sympistis chionanthi*, CT: Windham Co., Pomfret, adult at light (UCMS).

**HOLOTYPE** male (Fig. 1) IA: Boone Co., Little Bluestem Prairie [41°53'52N, 93°52'10"W], [larva] 29 May 2010, Mary Jane Hatfield, 051B-B10, [adult emerged] 9 September 2010, host: *Triosteum perfoliatum*; Genitalia CNC slide # ♂ 16516; Barcodes of Life Project # CNCLEP 81921, leg removed, DNA extracted. Deposited at UCMS, Storrs, Connecticut, USA. **Paratypes (adults).** (22 males, 23 females) **Iowa:** Polk Co., Snyder Farm [41°46'23.51"N, 93°29'21.38"W] [larva] 316 May 2009, Mary Jane Hatfield; DLW Lot: 2010E96, emerg: 29 August 2010, Host: *Triosteum perfoliatum*, (1 ♂) (UCMS); Polk Co., Snyder Farm [41°46'23.51"N, 93°29'21.38"W] [larva] 30 May 2009, Mary Jane Hatfield, emerg: fall 2009, Host: *Triosteum perfoliatum*, (1 ♀) (UCMS); Polk Co., Snyder Farm [41°46'23.51"N, 93°29'21.38"W], 9 May 2010 [larva], 004-P10, Mary Jane Hatfield, [adult] found dead 9 September 2010, Host: *Triosteum perfoliatum* (1 ♂) (UCMS); Boone Co., Little Bluestem Prairie [41°53'52N, 93°52'10"W], May 2010 [larva], Mary Jane Hatfield, 051-D-B10, emerged 19 September 2010, Host: *Triosteum perfoliatum* (1 ♀) (UCMS); Boone Co., Little Bluestem Prairie [41°53'52N, 93°52'10"W], 29 May 2010 [larva], Mary Jane Hatfield, 051C-B10, 13 September 2010 [emerged], Host: *Triosteum perfoliatum* (1 ♂) (UCMS); Boone Co., Little Bluestem Prairie [41°53'52N, 93°52'10"W], 29 May 2009 [larva], Mary Jane Hatfield, 051B-B10, emerged 11 September 2010, Host: *Triosteum perfoliatum*; Barcodes of Life Project # CNCLEP 81922, leg removed, DNA extracted; Genitalia Slide CNC #16517 ♀ (UCMS); Boone Co., Little Bluestem Prairie, 41°53'53.83"N, 93°52'10.31"W, Sept. 2011, MJ Hatfield coll. (3 ♂) (2 UCMS, 1 ISIC); Boone Co., Little Bluestem Prairie, 41°53'53.83"N, 93°52'10.31"W, prairie remnant edge, larva May 5 2013, MJ Hatfield coll., 010E-B13 (3 ♀) (1 UCMS, 2 ISIC); Boone Co., Little Bluestem Prairie, 41°53'53.83”, N, 93°52'10.31"W, prairie remnant edge, larva May 5 2013, MJ Hatfield coll., 010C-1-13 (1 ♂) (ISIC); Kossuth Co., Algona, larva 20 May–14 June 2013, emerged 5 Sept. 2013, Matt Kenne coll. (1 ♀) (UCMS); Kossuth Co., Algona, larva 20 May–14 June 2013, emerged 10 Sept. 2013, Matt Kenne coll. (1 ♀) (UCMS); Kossuth Co., Algona, larva 20 May–14 June 2013, emerged 30 August 2013, Matt Kenne coll. (2 ♀) (UCMS); Kossuth Co., Algona, larva 20 May–14 June 2013, emerged 2 Sept. 2013, Matt Kenne coll. (1 ♂) (ISIC); Kossuth Co., Algona, larva 20 May–14 June 2013, emerged 3 Sept. 2013, Matt Kenne coll. (1 ♂) (NDSU); Kossuth Co., Algona, larva 20 May–14 June 2013, emerged 3 Sept. 2013, Matt Kenne coll. (1 ♀) (NDSU); Kossuth Co., Algona, larva 20 May–14 June 2013, emerged 6 Sept. 2013, Matt Kenne coll. (1 ♀) (ISIC); **Illinois:** Champaign Co., Mahomet, reared ex larva, 9–14 Oct. 1976, 27 Aug. 1976, 13 Aug. 1981, G. Godfrey coll. (7 ♂, 5 ♀) (CUIC); Cook Co., Elk Grove, [adults] bred 20–24 Aug. 1941 and 26–28 Aug. 1942, A.K. Wyatt (1 ♂, 3 ♀) (CUIC); **Minnesota:** Houston Co., Perkin’s Bluff Prairie, 43°47'8.85"N, 91°36'58.63"W, larva 11 May 2013, emerged 5 Sept. 2013, Mary Jane Hatfield coll. (3 ♀) (1 CNC, 1 CHC, 1 NMNH); Houston Co., Perkin’s Bluff Prairie, 43°47'8.85"N, 91°36'58.63"W, larva 11 May 2013, emerged 5 Sept. 2013, Mary Jane Hatfield coll. (1 ♂) (NMNH); Houston Co., Perkin’s Bluff Prairie, 43°47'8.85"N, 91°36'58.63"W, larva 11 May 2013, Mary Jane Hatfield coll. (4 ♂) (1 CNC, 1 CHC, 2 UCMS). **Paratypes (larvae). Iowa:** Polk Co., Snyder Farm [41°46'23.51"N, 93°29'21.38"W], Col: 29 March 2012 [larva], 9 May 2012 [preserved]; Mary Jane Hatfield, Host: *Triosteum perfoliatum* (UCMS); Story Co., Harker Savannah [41°54'6.73"N, 93°30'31.21"W], Col: 29 April 2012 [larva], 9 May 2012, [preserved] Mary Jane Hatfield, Host: *Triosteum perfoliatum* (UCMS); Story Co., Harker Savannah [41°54'6.73"N, 93°30'31.21"W], Col: 29 April 2012 [larva], 9 May 2012 [preserved]; Mary Jane Hatfield, Host: *Triosteum perfoliatum* (UCMS); Winneshiek Co., [43°27'51.96"N, 91°38'15.73"W], Col: 15 May 2012 [larva], 18 May 2012 [preserved], Mark Leoschke, Host: *Triosteum perfoliatum* (UCMS); Allamakee Co., [43°25'16.65"N, 91°16'54.53"W], Col: 23 May 2012 [larva], 24 May 2012 [preserved], Mark Leoschke, Host: *Triosteum perfoliatum* (UCMS); Boone Co., Danielle Wirth property (oak savannah) [41°52'11.29"N, 93°52'55.10"W], Col: 22 May 2010 [larva], 28 May 2010 [preserved], Mary Jane Hatfield, Host: *Triosteum perfoliatum* [dissected] (UCMS).

**Figures 13–18.**
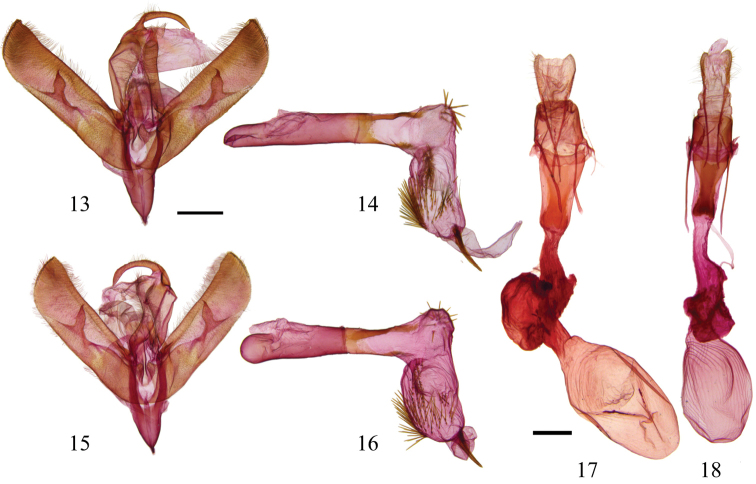
*Sympistis forbesi* and *Sympistis chionanthi* genitalia **13** *Sympistis forbesi* HOLOTYPE male, IOWA: Boone Co., Little Blue Stem Prairie, Genitalia CNC slide # 16516 ♂; scale = 1 mm **14** aedoeagus, same data **15** *Sympistis chionanthi* male, MANITOBA, Cartwright, Genitalia CNC slide # 16515 ♂ **16** aedoeagus, same data **17** *Sympistis chionanthi* female, SASKATCHEWAN, 8 mi NW Stewart, 1800’, Genitalia CNC slide # 13192 ♀; scale = 1 mm **18** *Sympistis forbesi* paratype female, same data as male, Genitalia CNC slide # 16517 ♀.

#### Etymology.

We name the species after William T. Forbes, North America’s premier lepidopterist over a 40-year period from 1920 to 1960. Forbes’ understanding of the species and higher-level taxonomy of eastern Macrolepidoptera was extraordinary, with the vast majority of his taxonomic decisions standing the test of time (and additional data). His four-volume treatise on the *Lepidoptera of New York and Neighboring States* remains the definitive work on eastern moths, especially for most Microlepidoptera.

#### Diagnosis.

**Adult.** *Sympistis forbesi* averages slightly smaller than *Sympistis chionanthi*. The scales over the thorax are smaller, more densely packed. In most individuals there are fewer white scales on the thorax and forewing: e.g., the costal margin, wing base, and orbicular and reniform spots have fewer white scales than most individuals of *Sympistis chionanthi*. Additionally, the anal dash is often more J-shaped and the fringe is only faintly checkered, lacking the pure white scales seen in many *Sympistis chionanthi*. In the hindwing, there is a more distinctive terminal line and the apex tends to have more black scales extending onto the fringe. The rami of male antennae through the basal half of the antenna average 0.50–0.65 mm in *Sympistis forbesi*, and 0.55–0.70 mm in *Sympistis chionanthi*. **Larva.** The larva provides unambiguous morphological characters that allow recognition of this new species. The last instar is mostly green with a reddish dorsum (red coloration is added through mid to late instars); there are no black or brown markings as in *Sympistis chionanthi* (in particular, the black subdorsal stripe characteristic of *Sympistis chionanthi* is absent from all instars of *Sympistis forbesi*). Body smaller, more elongate and modestly tapered at both ends, especially relative to the robust habitus of *Sympistis chionanthi*. Head width of ultimate instar of *Sympistis forbesi* 2.5–2.8 mm; head width of *Sympistis chionanthi* 3.0–3.2 mm. Spiracular height consistently smaller in *Sympistis forbesi* compared to *Sympistis chionanthi* – mean spiracular heights of A1–A6 are 0.30 and 0.36 mm, respectively. Mean crochet number of *Sympistis forbesi* on A3–A6 and A10 are 17, 19, 20, 21, and 20; mean crochet number of *Sympistis chionanthi* on A3–A6 and A10 are 27, 28, 32, 32, and 33.

#### Description of adult.

**Male.** Forewing length: 14.5–16 mm (n=23, reared from wild larvae). Ground color warm gray. **Head.** Antenna biramous; rami approximately 0.50–0.65 mm through basal half of antenna. Forward-facing tuft of scales just above faint black line between eyes. **Thorax.** Gray, medial prothoracic tuft, edged with black, preceded by conspicuous transverse black line. Black edging of tuft continues laterad to wing base. Tegula steely gray, indistinct thick band of dark scales at back. Legs with mix of dark and light scales. Tarsi dark brown or black. **Forewing.** Thin, smoothly curved basal and antemedial lines. Thickened antemedial line tapering to inner margin. Orbicular spot gray centrally and pale gray peripherally, thinly edged with black. Medial line ill defined; field proximal to reniform spot with numerous dark scales, forming two dark fascia along costa above orbicular and reniform spots. Black line or open triangle in position of claviform spot. Postmedial line running parallel to medial line, connecting to base of reniform and looping around toward margin, finally connecting to dark fascia along costa. Anal dash usually crisp, occasionally absent, subtended by sharp or diffuse black spot basad, forming J-shape. Subterminal line forming black fascia at costa, but otherwise pale gray, weakly developed to nearly obsolescent. Fringe weakly checkered, without white scaling. **Hindwing.** Pearly white with thin, crisp terminal line except at apex where diffuse field of black scales extends through fringe. Postmedial line obsolescent in males. **Abdomen.** Mixture of light and dark scales and hairs. Whitish scales along posterior margin of pregenital abdominal terga. **Male genitalia.** Valves elongate, nearly parallel sided with flat-topped projection from apex; bulbous clasper with claw-like apex that curves mesad; corona of fine setae of variable lengths. Juxta poorly differentiated. Uncas curved, gradually tapering, apex drawn into fine, curved spine. Saccus V-shaped, drawn into point anteriorad. Aedeagus cylindrical, variously sclerotized with vesica bearing approximately one dozen spines on elbow-bend and numerous longer, narrower spines over bulbous subapical region; terminus armed with single stout spine nearly 1 mm in length (as large as uncus).

**Female.** Forewing length: 14–16.5 mm (n=10, reared from wild larvae). Similar to male, but with substantially more fuscous scaling in submarginal region of hindwing; often with faint postmedial band. Antenna simple, without rami. **Female genitalia.** Posterior and anterior apophyses slender, elongate, ca. 2.5 × length of sclerotized portion of A8; lamella antevaginalis sclerotized, winged anteriorally and posteriorally, with posterior part about ostium bursae cleft and thus appearing somewhat flipper-like; anterior end less flared, ca. ½ width and only shallowly cleft, and more strongly sclerotized. Appendix bursae well developed; ovate corpus bursae ca. 2 × size of appendix bursae with parallel thickenings most evident posteriorad.

**Description of pupa** (Figs 28, 29). 16–19 mm long, 4.4–5.0 mm wide. Orange brown to deep chestnut brown, mostly smooth except for deeply pitted anterior portion of abdominal segments A4–A7. Primary setae extremely short, difficult to locate. Labial palpus visible, subequal to visible portion of profemur. Foreleg with cuminate apex, ending in abrupt spine. Proboscis extending just beyond antenna and midleg, nearly reaching end of wing. Labrum roughly shovel shaped with truncated apex. Eyepiece and frons ornamented with dense micro-ridging. Spiracular scars elongate, five times longer than wide. Cremaster ending in pair of minute thorn-like spines; cremaster deeply wrinkled and heavily sclerotized at base.

**Description of living final instar** (Figs 31, 32). Ground color sea to mint green with pink to red dorsum and pale longitudinal striping along sides of trunk; A8 modestly humped. Reddish dorsum composed of pink to pale red middorsal stripe flanked by darker red addorsal stripes; dorsal pinacula white. Mostly broken white pinstripe zigzags through D1 pinacula. Red dorsal area bounded by pale (green to white) subdorsal pinstripe. Two supraspiracular pinstripes edged below with darker green. Lateral stripe, greenish white, roughly equal to height of spiracles, extending along lower end of spiracles. Prolegs on A3 and A4 about half size of those on A5 and A6. Head pale to dark brown above, often with pink to reddish flush; labrum greenish white, shallowly rugose frons, gena with three whitish lines that anastamose about stemmata.

**Description of living early instars** (Fig. 30). All instars elongate, smooth, with numerous stripes; A8 modestly humped. First and second instars reddish brown and white, shiny; middorsal white stripe enlarged over anterior half of A8. Subspiracular white stripe thickened, enlarged to include each spiracle along trunk. All pinacula distinct, raised, brown black. First instar head width 0.15–0.16 mm. Second instar head width 0.40–0.56 mm. Third instar green and white, with brown-black pinacula; head width 0.90–1.00 mm. Fourth instar with small white pinacula as in final instar; head width 1.50–1.80 mm.

**Figures 19–23. F3:**
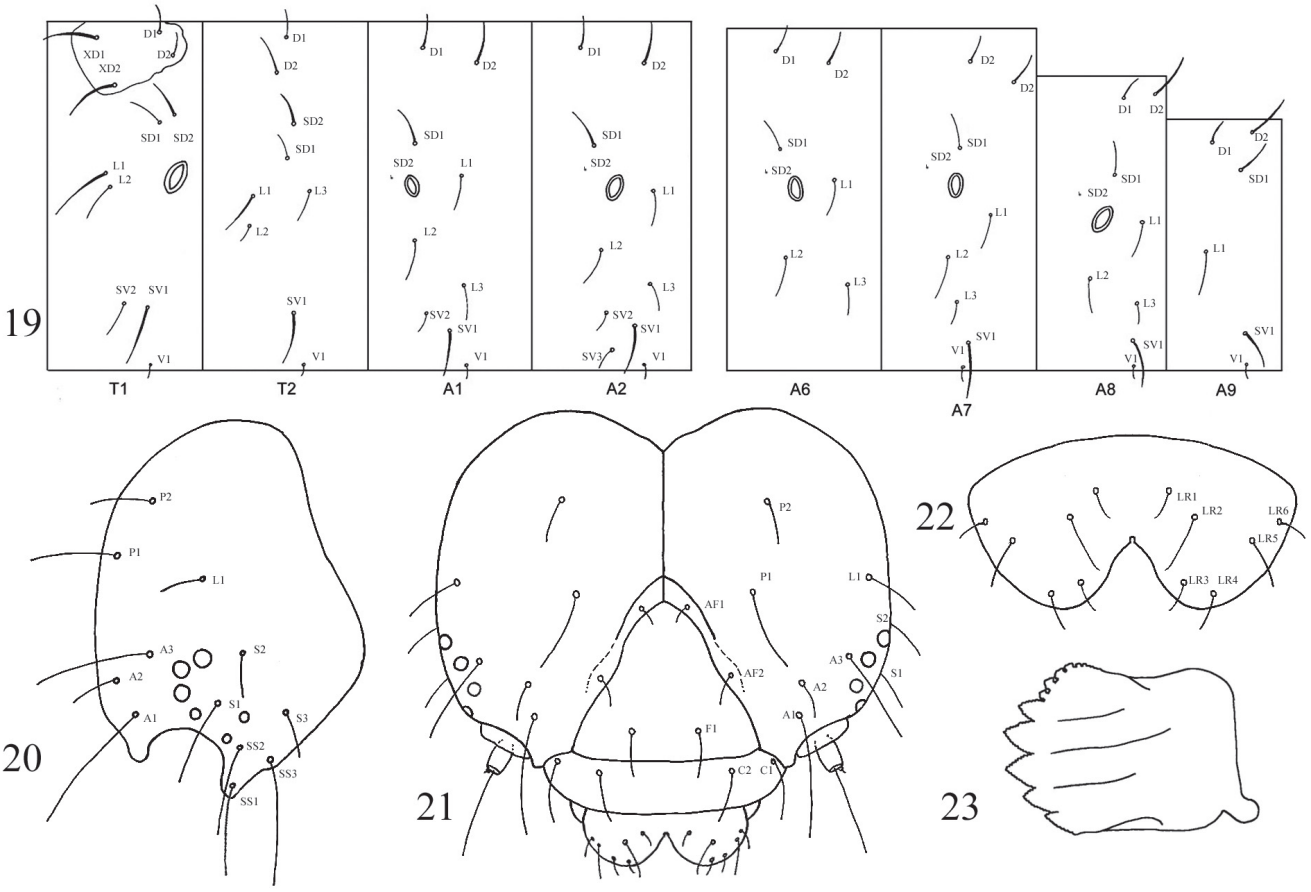
*Sympistis forbesi* larva. **19** chaetotaxy **20** head, lateral **21** head, frontal **22** labrum **23** mandible.

**Figures 24–27. F4:**
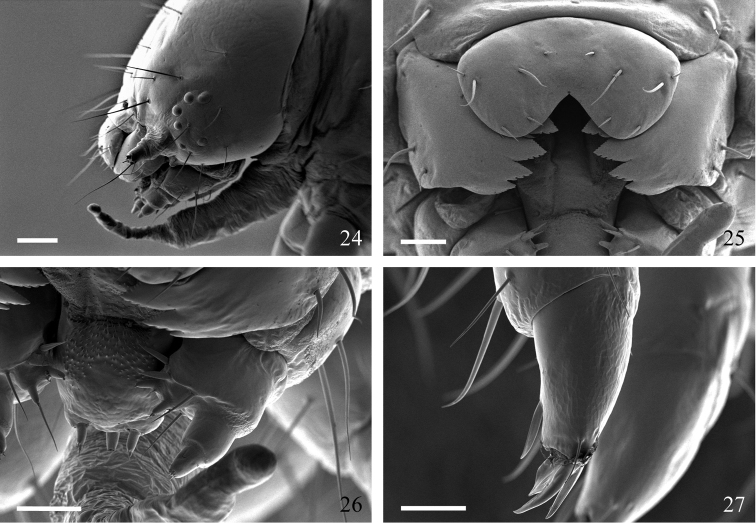
*Sympistis forbesi* middle instar. **24** head, lateral, with adenosma extruded; scale = 250 µm **25** labrum, mandibles, and oral cavity; scale = 100 µm **26** hypophryngeal complex (center left) and maxilla (center right); scale = 100 µm **27** prothoracic leg (note apical and subapical blade-like setae proximal to claws); scale = 100 µm.

**Description of preserved final instar** (Figs 19–27). **Head.** Texture microtuberculate. Width 2.5–2.8 mm. Field of brown aggregated spots on vertex, especially between P setae and L1. Second group of spots caudad of S3. P1 2 × length of P2. A1 longest seta on head (Figs 20, 21, 24). V-shaped medial cleft about 2/5 labral depth (Figs 22, 25). Spinneret short, subequal to labial palpus (Fig. 26). Mandibles simple, inner surface mostly smooth (Fig. 23). **Body.** Length 34–44 mm. Integument smooth; lightly sclerotized prothoracic shield and anal plate. Primary setae short, most approximately 2 × height of spiracle on same segment (Fig. 19). **Thorax.** SV1 longest seta on thoracic segments, ca. 3 × height of prothoracic spiracle and 1½ × height of SV2; spiracular height: 0.30–0.32 mm. Prothorax with SD1 and SD2 free from shield, positioned above spiracle. L1 3 × longer than L2 on T2 and T3. Meso- and metathorax with D and SD setae more or less vertically aligned (Fig. 19). Thoracic legs with apical and subapical blade-like setae proximal to claws (Fig. 27). **Abdomen.** Two SV on A1, three SV on A2. L1 directly behind spiracle on A1–A6 and A8, displaced ventrad on A7. D2 becoming increasingly procumbent towards caudal end of body (Fig. 19). D2 seta on A8 and A9 arising from slightly elevated and pigmented, rearward-facing wart. A10 with D and SD setae on rearward facing warts. Spiracular height of A1 through A6 0.28–0.32 mm; height on A7 0.26–0.28 mm; height on A8 0.32–0.35 mm. Crochet numbers on A3–A6 and A10 as follows: 16–18, 17–22, 19–22, 20–23, and 20.

#### Remarks.

We were unable to identify any consistent genitalic differences in either the male or female genitalia that distinguish *Sympistis forbesi* from *Sympistis chionanthi*. Our type series is based on reared material, as we know of no definitive structural or patterning characters that will assure certain identification of light-collected adults. Hence, we caution that features discussed in the diagnosis and description may be attributes more typical of reared (unflown) specimens. For example the slightly smaller size and darker coloration that we note above for *Sympistis forbesi* could be rearing artifacts.

#### Distribution.

Locally common in Midwest, especially prairies. Most commonly found in Iowa, Illinois, and Minnesota. Believed to be extirpated from eastern portion of range in New York and New Jersey. Given that the genus *Triosteum* occurs from southern Canada to Texas and eastward, it is probable that the range of the new species is more extensive than circumscribed here.

#### Biology.

So far as known larvae are specialists on members of the genus *Triosteum*, also known as horse-gentian or feverwort, of the family Caprifoliaceae. Nearly all our larval collections are from *Triosteum perfoliatum* (feverwort). We found a few larvae of what appeared to be the same species on *Triosteum aurantiacum* in Iowa. In the laboratory, larvae from *Triosteum perfoliatum* readily accepted and matured on *Triosteum aurantiacum*. In two separate instances, larvae were successfully reared to pupation on *Fraxinus* as well, a widely used host plant of *Sympistis chionanthi*. *Sympistis forbesi* larvae grew more slowly on *Fraxinus*, and maintained their typical green and pink coloration (MJH unpublished data, M. Keene personal communication). *Sympistis forbesi* is univoltine with a single generation that emerges, flies, and mates in late summer, mostly in early September. Females presumably lay eggs on or near the stems of *Triosteum*. Above-ground tissues of the host die and senesce over the winter. MJH has found first instars on unopened leaves that were just pushing forth from the ground in early March (in Iowa). Larvae complete their development by mid-June. Early instars feed exclusively on new leaves, principally of the apical meristem, before the leaves have had a chance to open and expand to full size. Where the moth is common and when collections are made through the first half the season, partially opened leaf fascicles often yield larvae that were not seen at the time of collection. Last instars also consume new leaves, but are content to feed on fully expanded leaves and flowers (Figs 34–36). All instars are cryptic in both color and habit. The late instars rest along a shoot head down, often near flowers, where their coloration is well matched to that of the stem and reddish-pink *Triosteum* petals and sepals. Densities can be high with more than a dozen larvae on a single shoot; on several occasions we noted cases where the larvae of *Sympistis forbesi* severely damaged the apical portions of their host plant. Prepupae form a slight cocoon below ground; the summer months are passed as a pupa.

#### Barcoding.

In a neighbor-joining tree based on J. D. Lafontaine’s unpublished barcodes for 137 North American oncocnemidine noctuids (representing 687 individuals), *Sympistis chionanthi* (n=7; CT, Quebec, Ontario, Alberta) and the new species (n=2; both Iowa) grouped together in a “cluster” separate from other North American *Sympistis*, and each taxon was reciprocally “monophyletic,” although the two groups differed by less than 1% from one another. In a second analysis, focused on “*Adita*-group” *Sympistis* that included 13 individuals from across North America, again the two *Triosteum* feeders grouped in their own cluster.

#### Discussion.

*Sympistis chionanthi* was described by J. E. Smith in Abbot and Smith 1797 based on a painting of the adult, caterpillar, pupa, and the host *Chionanthus virginica* Linn. (fringetree) (family Oleaceae) by Abbot. As is the case for all of taxa drawn by Abbot, there is no type specimen for *Sympistis chionanthi*. The common name “Grey O Moth” was given for the “O” shaped orbicular spot on each forewing. Abbot’s rendering of the larva depicts a robust caterpillar that is pale brown laterally, shaded with darker brown dorsally, and bears a thick black subdorsal stripe, a slight stripe behind the head and a moderately humped A8 segment. His illustration is undoubtedly a match for the current-day *Sympistis chionanthi* caterpillars from *Fraxinus* (Fig. 33). Despite not having any type material for *Sympistis chionanthi*, we are confident in our assessment that the original species description agrees with the current understanding of *Sympistis chionanthi* and that *Sympistis forbesi* represents a distinct species.

*Sympistis chionanthi* was described as being very rare in Georgia (Abbot and Smith 1797). There are no recent reports of *Sympistis chionanthi* living in Georgia (J. Adams pers. comm.), and even in North Carolina it is an extremely rare mountain taxon (Bo Sullivan pers. comm.).

Although the adults of *Sympistis forbesi* and *Sympistis chionanthi* are difficult (and sometimes impossible) to distinguish even upon dissection, their larvae are distinct in size, coloration, habitus, and life history. Presumably these coloration and morphological differences reflect, at least in part, the structural differences in their preferred hosts. *Triosteum* is an herbaceous perennial that dies back to the ground each winter; *Fraxinus* and *Chionanthus* are trees. The brown, bark-like coloration of late instar *Sympistis chionanthi* is suggestive that larvae rest off of foliage by day and perhaps even near the ground along the trunk or off the host in leaf litter. We know of no brown noctuoid larvae that rest on foliage by day, and many, like *Catocala* Schrank, *Melipotis* Hübner, and *Zale* Hübner, may wander far from the foliage when not feeding. The coloration of last instar *Sympistis forbesi* (Figs 32, 33) is reflective of its preferred resting site: the green stems of feverwort. Likewise, it is our guess that the less robust body, smaller prolegs, and reduced crochet hook number of *Sympistis forbesi* reflect the fact that larvae rest adjacent to suitable foliage. By contrast, the caterpillars of *Sympistis chionanthi* on a mature ash or fringetree may well have to traverse meters in search of suitable food each night. Surprisingly, no differences in mandible morphology of the two sister taxa were noted.

Despite the differences between the hosts of *Sympistis forbesi* and *Sympistis chionanthi*, the plants share secondary metabolites which may elucidate how the ancestral host plant switch was able to occur. Triosteum are members of the Caprifoliaceae, whereas the hosts of *Sympistis chionanthi* (*Chionanthus* and *Fraxinus*) (Troubridge 2008, Robinson et al. 2012) are both Oleaceae. Both families are known to contain iridoid glycosides (Bowers 1991, Seigler 1998, Jensen et al. 2002, Lee et al. 2010). While larval hosts are known for only a small fraction of North American *Sympistis* (Troubridge 2008), at least among the known hosts, plants with iridoid glycosides figure prominently (Wagner et al. 2011). In western North America, *Penstemon*, in particular, well known to have iridoids (Stermitz et al. 1988, Krull and Stermitz 1998), supports numerous *Sympistis* species (DLW unpublished data).

Prior to Troubridge’s (2008) oncocnemidine revision, *Sympistis chionanthi* was classified in the monobasic genus, *Adita* Grote, 1874. Troubridge synonymized the genus into *Sympistis* and regarded *chionanthi* to be a highly derived species within *Sympistis* related to the *Sympistis dentata* species group. In addition to the species that we describe here, there may be additional cryptic species in collections sorted as “*Adita chionanthi*.” George Godfrey and Tim McCabe have beaten caterpillars of an “*Adita*” group species from *Symphoricarpos* Duhamel in the Upper Midwest. Images taken by Godfrey of these larvae closely approach those of *Sympistis chionanthi*, but differ in having more gray in the ground color and some brick red over the dorsum. Unfortunately, neither McCabe nor Godfrey reared adults or preserved larvae. John Franclemont collected a large series of “*chionanthi*” in Montana and reared an ex ova cohort on *Fraxinus*. Based on the number of pinned specimens, larval photographs, and genitalic dissections it seems likely that Franclemont believed the Montana populations might represent a new species. Adults average larger and brighter than material from eastern North America. Three individuals of “*chionanthi*” from nearby Alberta, also included in the barcoding dataset, clustered separately from the eastern individuals. Given the above, we caution that our figured male and female genitalic preparations (Figs 15–17) for *Sympistis chionanthi* are from central Canada; without larval or genetic data, we cannot with certainty know that these are nominate *Sympistis chionanthi*. We were unable to find consistent differences in male or female genitalia in *Sympistis chionanthi* (representing five states and provinces), or between *Sympistis chionanthi* and *Sympistis forbesi*. If our findings about the differences between *Sympistis chionanthi* and *Sympistis forbesi* are indicative for other members of the species group, or *Sympistis* more widely, larvae, life history data, and molecular data will be needed to tease apart the biological species in this complex.

Part of our interest in the new species derives from our desire to document instances where rates of phenotypic evolution in Lepidoptera differ markedly among life stages. For example, in *Acronicta* Oschenehimer and some notodontid genera (e.g., *Datana* Walker and *Schizura* Doubleday) larval phenotypes differ substantially among related species that are otherwise difficult to determine using external and genitalic features of the adults. Adults of *Acronicta hastulifera* (J. E. Smith) and *Acronicta dactylina* Grote are sometimes impossible to separate by eye or dissection, but each has a distinctive larva that readily distinguishes the second to final instars of both species (Wagner et al. 2011; Schmidt and Anweiler unpublished data). Conversely, plusiine caterpillars are remarkably undifferentiated relative to their adults, and often require microscopic examination even to make generic and tribal assignments (Crumb 1956, Lafontaine and Poole 1991). The adults of *Sympistis forbesi* and *Sympistis chionanthi* are mixed in collections that we have examined (under the latter name). By contrast, their larvae, are immediately distinct, with the coloration of each approximating that of the stem-color of their primary hosts: feverwort (*Triosteum*) for *Sympistis forbesi* and ash (*Chionanthus* and *Fraxinus*) for *Sympistis chionanthi*. With careful morphological analysis, it may be possible to quantify these differing rates of phenotypic evolution within and between species using newly developed phylogenetic techniques (e.g., Adams 2013).

**Figures 28–29. F5:**
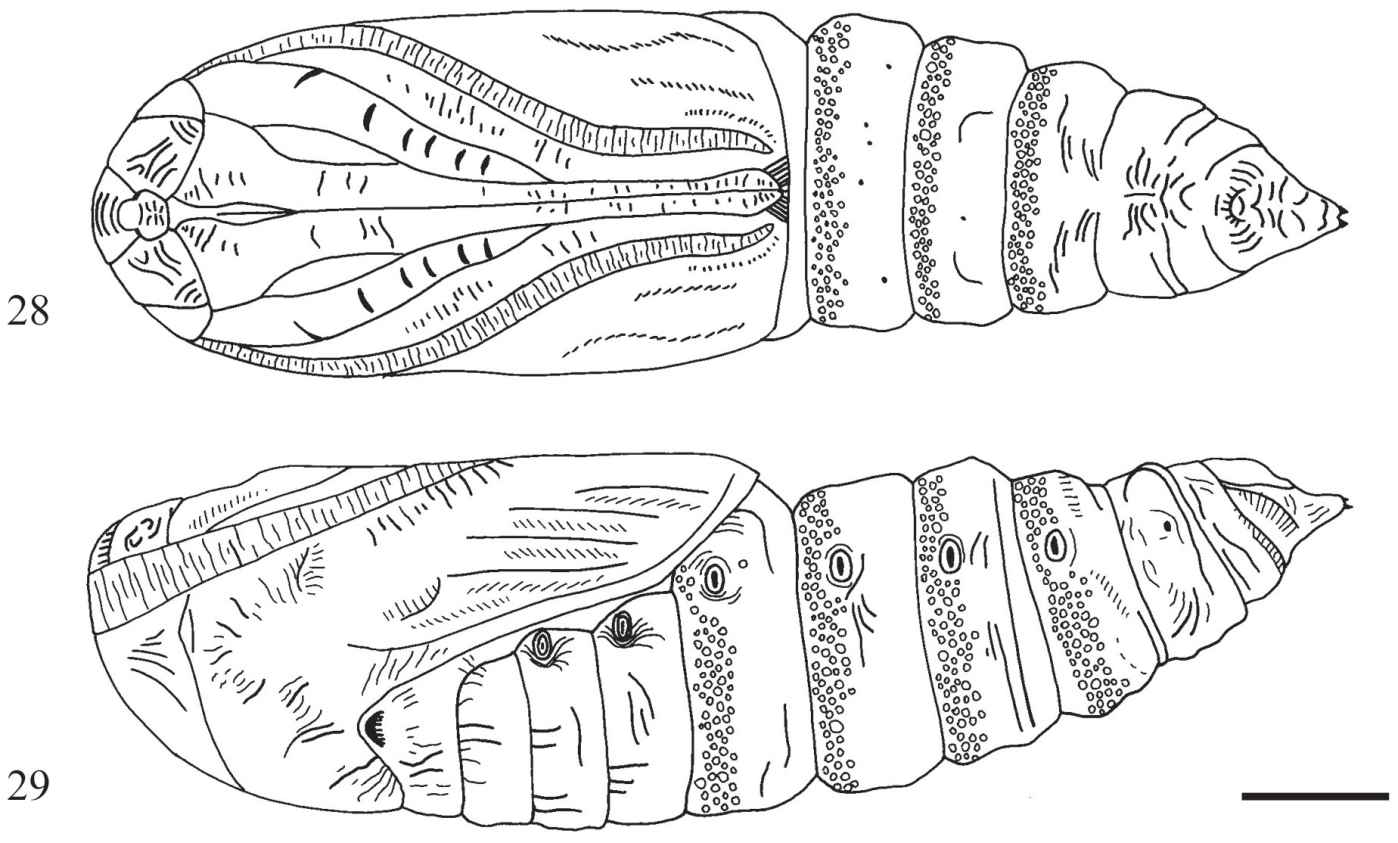
*Sympistis forbesi* pupa **28** ventral **29** lateral.

**Figures 30–33.**
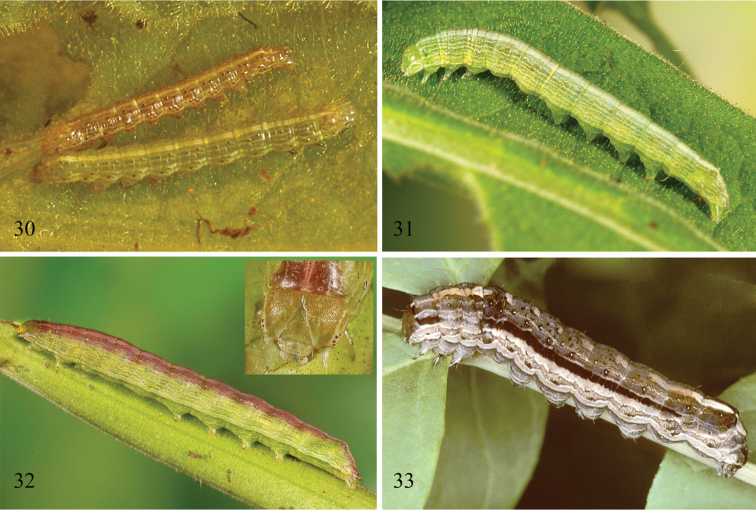
*Sympistis forbesi* and *Sympistis chionanthi* larvae. **30** *Sympistis forbesi* second (upper) and third (lower) instars. IA: Boone Co., Little Blue Stem Prairie, May 2011, ex *Triosteum perfoliatum* **31** *Sympistis forbesi* middle instar, same collection data **32** *Sympistis forbesi* mature last instar, same collection data **33** *Sympistis chionanthi* mature last instar, NY: Albany Co., Albany, female fall 1995, ex ova reared on *Fraxinus americana*, DLW Lot: 1996F32.

**Figures 34–36.**
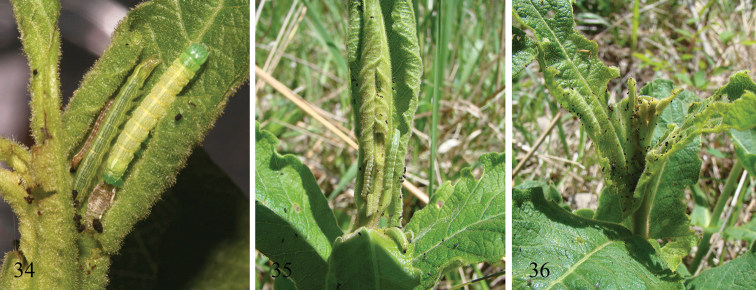
*Sympistis forbesi* IA: Boone Co., Little Blue Stem Prairie, May 2011 on *Triosteum perfoliatum* **34** three larvae secreted in a leaf axil; note frass accumulation **35** larvae on new spring leaves; note two larvae on new leaf bundle and one on foreground leaf **36** last instar on a flower of *Triosteum perfoliatum*, matching the color of the flower and petioles.

## Supplementary Material

XML Treatment for Sympistis forbesi
